# Challenging “The Hands of Technology”: An Analysis of Independent Living for People with Intellectual Disabilities

**DOI:** 10.3390/ijerph19031701

**Published:** 2022-02-02

**Authors:** Joan Moyà-Köhler, Miquel Domènech

**Affiliations:** 1 Facultat Pere-Tarrés, Universitat Ramon Llul, 08021 Barcelona, Spain; jmoyak@peretarres.org; 2 Departament de Psicologia Social, Universitat Autònoma de Barcelona, 08193 Barcelona, Spain

**Keywords:** intellectual disabilities, technology, independent living, Science and Technology Studies

## Abstract

Technology has been holding out the promise of facilitating greater autonomy and improving care for people in a situation of dependency. This trend is expected to grow and this is happening precisely at a time of expansion of the so-called Independent Living paradigm. In this context, however, disability activists are generally suspicious of approaches based on being “left” in the hands of technology. They instead advocate for “subordinating hands” to their ability to decide, a principle that stands in tension with the field of intellectual disability, where individuals are perceived as intrinsically unable to make “good decisions”. Therefore, the aim of this paper is to provide insight into the uses and developments of technologies with regard to care and autonomy for people with intellectual disabilities. By ethnographically examining the case of a specific technology; QR (quick response) codes in the context of an independent living service, and in the framework of Science and Technology Studies and Disability Studies, the paper reveals the role and possibilities of care and autonomy technologies for people with intellectual disabilities. Based on these findings, and by thinking from what we could define as “within a sociotechnical assemblage”, this paper aims to rethink the ways in which technologies for independent living can be used in the field.

## 1. Introduction

Video-call assistance, telecare, online tutorials, digital shopping lists, QR (quick response) codes, smartphone payments..., for years now, technology has been holding out the promise of facilitating greater autonomy and improving care for people in a situation of dependency. This changing scenario has been accelerated by the outbreak of the global COVID-19 pandemic [1,2]. The trend is expected to persist and grow in the coming years [3], especially in the disability sector, where future imaginaries of care have been built on technological grounds for some years now.

The field of intellectual disability has not been immune to such rapid changes in the ways of providing care. For instance, in Spain it was observed that 66.7% of personal assistance was provided telematically during the outbreak of the pandemic [4] and there were similar findings for many support services around Europe [5]. Thus, information and communication technologies (ICTs) have been seen as the best tool to continue providing logistical and emotional support to individuals and families in this context, but they have also entailed substantial restructuring over time. 

This change in the ways of providing care has emerged precisely at a time of expansion in this field of the so-called Independent Living paradigm, which was created more than 50 years ago through the activism of the Independent Living Movement (ILM) and anti-institutionalization movements, which, under the slogan “nothing about us without us”, voiced criticism about the ways in which scientific and technological developments were happening [6]. From there on, activism in the field has provided stimulating reflections on the very idea of disability and has been demanding the right to a life on equal terms. On the one hand, it has called for structural changes to foster accessibility in the public space and, on the other, has insisted on social and technical support for people’s autonomy [7].

This last point is particularly interesting regarding the present special issue and its title, because the “in the hands” part of “In the hands of technology” has been precisely one of the battlefields of activism in the disability arena. The ILM has been particularly active in warning against the harmful effects of being in the hands of others (the state, informal caregivers, or invasive technologies, for example). From here, the theoretical and practical developments in the field of Independent Living have gone hand in hand with the promotion of methods whereby technological and scientific developments are subordinated to the decisions of the concerned individuals themselves. The “nothing about us without us” motto imposes a sort of “WITH the hands of technology”, a critique of the predominantly biomedical models of subordination and a campaign for these people to regain control over their own lives in whatever affects them. It is not about being in the hands of anyone, but about being given hands that are subordinate to their ability to decide what and how in their lives.

But it is precisely this postulate, the one that links self-determination to the ability to decide, which is in tension when we enter the field of intellectual disability. The modern idea of the individual who, ultimately, should decide for himself/herself how they are to be supported, is exactly what is still a matter of discussion regarding the subject of people with intellectual disabilities, which is immersed in discourses, structures, and practices that continue to infantilize these groups [8], presupposing an intrinsic inability to make “good and informed” decisions.

From here, the methods for implementing the independent living paradigm for people with intellectual disabilities have been somewhat idiosyncratic with respect to the physical disabiled community [9]. Thus, at a time when new care technologies are expanding within the sector, it is especially relevant to pay attention to their uses and developments and to focus on how they are designed and implemented.

Within the framework of Science and Technology Studies (STS) and Disability Studies (DS), and after explaining the methodology used for this research, this paper presents the results obtained from a study of the use and implementation of ordinary technologies [10]. For this purpose, it focuses on an ethnographic study of a specific technology: QR codes in the context of an independent living service for people with intellectual disabilities as implemented by the “Fundació Catalana de la Síndrome de Down” (the Catalan Down Syndrome Foundation, hereinafter, FCSD) through the “Me’n vaig a casa” (I’m going home) program.

Based on this study, the paper shows how a single technology can be more or less successful in the field of intellectual disability depending on the logic (enabling or prosthetic) from which it is incorporated. A prosthetic logic poses limitations for people whose decision-making capacity is under discussion, while an enabling logic opens new avenues for this collective’s independent living.

These results allow us to question the very notion of care and autonomy in the field, and at the same time they help to rethink the development of technologies addressed at people with intellectual disabilities. Drawing from this, our proposal is to imagine and conceive the progress of ICTs not so much from an “in the hands” or a “with the hands” perspective, but from a “within a sociotechnical assemblage” one.

## 2. Theoretical Framework

It is important at this point to briefly address the complex relationships between disability, care, autonomy, and technology. To do so, we will present some of the theoretical approaches that intersect in this study; on the one hand, Disability Studies and especially its Social Model and, on the other, Science and Technology Studies (STS), especially from the Actor Network Theory (ANT).

First, it is important to contextualize the idea of care and its connection with autonomy. Our aim is to avoid modern notions of autonomy that consider it to be a certain mode of individual rationality and self-determination [11]. Instead, we propose a notion of autonomy based on the idea of interdependencies, so that it is completely crossed by the notion of care—understood here from an Ethics of Care point of view as everything that is done or arranged to sustain life [12]. This way of understanding autonomy, and broadening the view of care work, seems indispensable for apprehending relational frameworks and thinking beyond the rational, autonomous, and independent subject presented by modernity. Care is therefore transversal to all subjects, who are always dependent—and therefore interdependent among others—on a web of care relations that extend to, as we shall see, heterogeneous human and non-human elements. In the case we are presenting here, these networks are responsible for the emergence of a certain sort of autonomy.

In order to talk about autonomy, it is important to point out that the field of disability has witnessed major changes in recent years in terms of its conceptualization, especially from the so-called Social Model of Disability, which emerged from social movements in the field and is based on successful collective identification with the concept of “disability” and experiences of discrimination against “disabled” people [6]. This theoretical model is based on the conceptual distinction between the biological, impairment, and the social, disability. Thus, disability comes to be problematized as a situation of social and political exclusion and hence the need to defend its personal right to decide [13,14].

The development of the Social Model of Disability was particularly influential, giving rise to certain basic principles such as the promotion of human and civil rights, mutual aid, empowerment, responsibility for one’s own life, the right to take risks and to live as part of the community [15], and allowed central issues to be placed on the disability agenda, such as the importance of self-help, user-driven services and political campaigns to achieve legal changes in the field [16].

These demands revolve around two main campaign areas: on the one hand, direct action on the environment and, on the other, access to the support needed to develop self-sufficiently [17,18].

As we have already mentioned, however, the field of intellectual disability is a somewhat complex one in which to consider some of these proposals. The infantilizing discourses on people with intellectual disabilities [19], as well as the idea of dealing with an irresponsible collective that lacks certain capabilities [20,21], have made it difficult to align with the Independent Living Movement’s proposals, which often advocate the ultimate right of individual decision [22].

Although the field of intellectual disability presents some clear limits in terms of the modern ideal subject, much of the literature encourages us to think about this subject in different ways. Authors such as Héléne Mialet [23], taking Stephen Hawking as an example, give us a glimpse of how there exists behind Hawking’s “marvelous mind” a whole series of socio-technical elements, practices, and expert knowledge—in other words, a great amount of invisible care and support work—that enables this “marvelous mind”. Hence, reasoning is not something detached from the world; we need a network of people, technologies or institutions to be able to do so. Thus, autonomy, also in the field of intellectual disability, is not only a certain capacity to decide, but a certain capacity to relate [24].

Therefore, this paper approaches the complex relationships between disability, autonomy, and technology from what some authors have called the “toolbox” provided by the Actor-Network Theory (ANT) [25]. This approach can be used to complexify the analysis and add new and necessary dimensions to the Disability field [26].

By attending to the Actor-Network Theory it is possible to add a further twist to the social shift that the Social Model paradigm represents. According to Galis:

“*This initial social approach of disability lacked a critical link between bodily experiences of disability, the development of social policies and the configuration of technology (and the built environment)*”.

([27], p. 828)

This symmetrical approach makes it possible to correlate the ability-disability dichotomy, described by the Social Model, with all those dichotomies that are part of the core-set of modern thought, such as nature-society, agency-structure, or subject-object, avoiding the reification of the two poles. 

As authors such as Moser [28] illustrate, in order to understand how a particular autonomy of a subject is shaped, we cannot start from his or her ascription to one of the two poles mentioned above. People are always immersed in socio-technical networks, in which prostheses, bodies or technical gadgets allow autonomous subjects to emerge. Agency is thus the product of established connections between elements that, on their own, cannot be considered to be responsible for actions. From here, Actor-Network Theory turns the notion of “agency” as a purely human trait and shows us how any specific action always has to do with all of the possible actors involved [29]. Thus, we can only attribute provisional “actor” roles, which are always immersed in a process of competence exchange [30]. 

In other words, agency is distributed among the different human and non-human actors that are linked at a specific time [31], so we can talk of “distributed agency” [32], a view that we could use to explain how people become able or unable [28]. Hence, the notion of autonomy acquires a new meaning, which is especially interesting in the field of disability and regarding the very conception of what an independent life entails, dislocating disabilities from within people to their way of relating with their environment [33].

We must therefore pay attention to what is part of the human and non-human network, the actants [34] that participate in this process of heterogeneous engineering [35] that entails the configuration of a person with intellectual disabilities as autonomous.

Finally, one last aspect to be considered, and which is essential in this paper’s field of study, is linked to care and how ANT addresses this issue. There is no possible autonomy without the maintenance work that enables the network to function, to make it operative. In this regard, it is highly pertinent to think from Miriam Winance’s idea of adjustment:

“*Through adjustment, the person is transformed and formed. What he or she materially and emotionally is, what his or her body and world are, are shaped. First, through this adjustment, “an extended body” arises that is both defined (it takes a certain form) and transformable. This “extended body” refers to a common materiality and to common perceptions. And second, the person’s world is transformed, possibilities and impossibilities are created, some entities are included, others are excluded. The adjustment is at the same time shaping of the body and shaping of the world. The emergence of an action is also the emergence of “a being,” which means that the emergence of an action supposes the transformation and formation of the ties making up the person, his or her body, his or her world, the transformation of what the person is*”.

([36], p. 67)

For Winance, material stability is constituted through the strength of the connections; it is not a question of the quality of the material itself, but of the links, of the affection of the elements at stake. From here, this work is closely linked to efforts to analyze the ways in which care is articulated through the technologization of interdependencies that sustain autonomy [37,38]. From this perspective, technology is no longer an inert concept to which we give meaning, but rather an actualization of practices, knowledge, and effects through which concrete ways of being in the world and relating to others emerge [39].

## 3. Materials and Methods

This paper is mainly based on two large corpora of data from a research study carried out as part of two interrelated projects conducted between 2015 and 2021. The first formed part of the doctoral thesis of one of the authors of the text and the second is part of the project titled “Infrastructures for independent living: a participatory research to rethink housing, care, and community in times of pandemic” funded by Barcelona City Council. Both studies are based on ethnographic approaches, or focused ethnographies [40], to a direct personal support service for people with intellectual disabilities, the Fundació Catalana de la Sindrome de Down’s ‘Me’n vaig a casa’ independent living project.

The work during the thesis was done between 2015 and 2016 and consisted of monitoring eleven cohabitation units. During this period, participant observation was performed while accompanying the support staff in the day-to-day running of the service, at the different meetings to form and follow-up the cohabitation units, as well as at meetings with the user’s relatives and acquaintances. At the end of the fieldwork, a total of 28 interviews were conducted with users (20), support staff (6), and family members (2). The whole process was recorded and analyzed by producing an exhaustive field diary.

The second study, part of the project titled “Infrastructures for independent living: a participatory research to rethink housing, care and community in times of pandemic”, emerged following the outbreak of COVID-19. Between May 2020 and October 2021, four self-help groups instigated by the same service were followed-up during the months of lockdown and the subsequent gradual easing of measures (March–June 2020). Later on, twelve interviews with users (7) and support staff (5) and follow-up of the development of the same service (June–October 2021) were carried out. Again, the whole process was recorded and analyzed by producing an exhaustive field diary.

For the present study, we have gathered different elements from those field notes and interviews, which provide an account of the different moments involving some of the technological devices used to promote the users’ autonomy.

The interviews and groups were recorded and subsequently transcribed. These transcriptions were first analyzed through a process of thematic analysis, and together with the data noted in the field diary process, ended up forming what some authors define as a “dense description” [41]. Thus, this work was not a purely descriptive process but rather a reflective approach to the interactions and meanings that emerged in the field, given that the application of descriptive techniques and procedures led to elaborate speculation. This work ended up structuring the analysis.

Both studies followed the ethical guidelines of the universities from which the work was carried out and received their approval. All participants in the study signed informed consent protocols to guarantee their privacy and, consequently, all names have been duly anonymized in the present paper.

## 4. Results and Discussion

“Me’n vaig a casa” (MVAC) is an independent living service directly inspired by the principles of Supported Living [42] that appeared in the USA in response to the traumatic closure of residential institutions [43]. Organized by the Fundació Catalana de la Síndrome de Down, it is a pioneering service in Spain and has served as an inspiration for many other projects in the sector.

The proposal from the Fundació Catalana de la Síndrome de Down focuses on individualized planning based on support and personal assistance for people with intellectual disabilities in order to achieve their greater inclusion within the community, providing the necessary tools to develop their own life projects based on living in their own homes [44]. In order to make it easier for people to choose how and with whom they want to live, a series of meetings are held with the so-called “circle of trust”—people chosen by the users themselves to make decisions collegially, generally family members—and meetings between the service and the users. These meetings are used to draw up a Personal Attention Plan, which serves as a guide to coordinate support and set objectives for the user’s independent life.

The service prioritizes their clients’ wishes, while linking them to their well-being and safety, which is a caring adjustment [45] that synchronizes [21] the users’ will with the need for protection from risks in their daily lives. To some extent, what this way of running the service shows is a logic of care [46], whereby caregivers only intervene from a non-imposing position, but at the same time from an active and concerned position towards the people to whom this support is provided [47]. All of this is done by means of a set of human and non-human resources, in a concrete assemblage to enable their functionality [24]. 

The support provided by the service includes, on the one hand, a certain number of hours per week of accompaniment by a support professional and, on the other, a network of technical elements that are made available to users. 

Of the different technical support elements that the service provides there is one, QR codes, that can be analyzed here to highlight the precarious relationship between autonomy and care for people with intellectual disabilities. What follows is a demonstration of how certain technological configurations, even within the same tech device, can adjust in different ways the care and autonomy provided to people with intellectual disabilities.

Marta and Claudia, two MVAC users, were given a set of QR codes distributed all over the house: next to the router, on top of the microwave, on the pressure cooker, in the dishwasher... From our different ethnographic visits we were unable to elucidate what use was made of these codes, since no use made of any of them on any of the more than twenty visits we made. The users passed by the codes without paying the slightest attention to them.

After a while, Marta, when asked what these codes were and what they were used for, explained that they allowed access to tutorial videos with instructions for the use of various devices in the home. These codes were arranged around the apartment to explain how the different devices worked and hence make it easier for the users to safely use them on their own, thus facilitating their autonomy. The QR code was developed by a tech company and the service conceived them an extension of the user’s memory “*if you don’t remember how it works, the QR code works as an external memory*” (Laura, excerpt from the field diary in November 2016).

Indeed, there are several tools and gadgets that can be used, for example, to remember what to buy or how to pay, such as shopping lists and wallets with specific amounts of money to make the payment. With all these elements the service manages to fix volatile elements, such as memory, through its materialization [48]. This same way of articulating technology as we saw with the QR code.

Delegating these tasks to the QR meant, in principle, that the users did not need to ask the support staff for help each and every time, and could instead use the different devices by themselves to thereby gain more autonomy. It seemed like a great idea, but it was not. In practice, it did not work. Once they had learned how to use it, there was no need to watch the video again, as Laura, one of the support workers, explains here:

*“Now the truth is that they don’t use it much because it is no longer necessary, when they have used it a couple of times, it’s done, isn’t it? (…) Of course, now they know how to use it and they don’t watch the video, but well, the codes are still there”*.

(Interview with Laura, November 2016)

After the first weeks had gone by, the users’ needs changed, and it was easier, in case of doubt, to call the support staff than to try to use a code every time they needed something, or when one of the factors changed. The codes quickly became obsolete. In fact, not even the users themselves were aware of their presence and ended up asking the support staff to help them to solve problems that could easily have been solved with the video. 

What we see in this case is how the QR technology is designed in terms of an individual possibility of acting, and by this the technological device imposes a very specific script [49] in which the individual is only autonomous insofar as he/she is following the allowed course of action in a disciplined way. By not doing so, the technology fails to form a successful part of the network.

Two years after the end of this first ethnographic work in the Independent Living service, the outbreak of the pandemic in 2020 offered an interesting context in which to understand how technological assistance for people with intellectual disabilities was being reconfigured. To this end, follow-up in the form of virtual meetings with users and a set of interviews were planned.

From these encounters, the biggest surprise was to witness how this same device, the QR code, had taken on a wholly new meaning. The use of this technology, which until now had been restricted to indoors, began to spread widely, especially to bars and restaurants, where its use became mandatory, replacing traditional printed menus in order to prevent COVID-19 transmission. 

This issue first arose at one of the meetings between users and some of the support staff during lockdown. At this virtual meeting in June 2020 Elena, one of the users, said the following:

*“Elena (user): the menu had like a QR code, and I took a picture and then the menu popped up… then you could choose what you want*.

*Monica (support staff): OK… but for example there is no menu to look at, you have to look at it on your mobile phone, right?*

*Elena: With the QR I took a picture with my mobile phone and there we could look at the menu and choose what we wanted*.

*Monica: Look Elena, you are very modern with QR… Tell me about your experience with QR. Because you talk about QR like it’s commonplace… explain what QR is and why you have so much experience with it, because that is very interesting. Because for example now we’re in this lockdown situation, aren’t we? And you for example have just arrived or started to make your independent life and suddenly… you find yourself there… So, what is it with the QRs you had, can you say?*

*Elena: We had QR stickers in the kitchen, for example. I put the mobile phone or the tablet, whatever it was, next to the QR and I saw step by step how to connect the WIFI at home. We had the QR code, for example, in the kitchen, and…for example, the day I went to cook, I used the QR and it showed all the steps. So… it was very good. It also explained how to put on a washing machine or a dryer. At that time, I was living with a roommate*.

*Monica: [to Elena] Yes, but when the two of you started living there, you were told how the QR works…*

*Elena: Yes, yes.*

*Monica: [to the whole group] That’s why she now sees a QR and she already knows. Now she can tell her parents, for example*.

*Elena: Yes, but I also go to the bar alone”*.

(Group meeting of “Me’n Vaig a Casa”, June 2020)

Therefore, it is not just a matter of external memory, by looking at it in these terms, we are unable to fully grasp its possibilities. Here technology is redefining people with intellectual disabilities: the person is able to sustain his or her meaningful spaces through a device that allows them to connect with and relate to their environment. 

The last sentence in the previous excerpt, when the user points out that she could “go alone”, highlights two very significant considerations. First, it shows the importance given in the field to the possibility of making the user emerge as the main performer of an action. Second, it reveals that the QR enables certain autonomy. But this autonomy is a precarious one, as we can see in the case of Andrés, who did not know how to use the codes:

*“When I go [to a restaurant] my mother knows how to use a cell phone to check it [the menu card], I don’t know very much about that”*.

(Interview with Andrés, September 2021)

In one of the subsequent interviews with another user, the case of QRs and their utility came up again. Francisco explained that he had learned to use the code within the MVAC context, and not only did he know how to use it, but had had the opportunity to explain to his mother how this technology was supposed to be used:

*“Interviewer: Because you already learned how to use it in the apartment where you lived independently, didn’t you?*

*Francisco: Yes, so I told my mother.*

*Interviewer: Did you tell her how to use QRs?*

*Francisco: Yes.*

*Interviewer: Wow, that’s good, isn’t it? And now she also uses it (…) for example, where did you use it?*

*Francisco: In a bar, and with my sister-in-law’s parents”*.

(Interview with Francisco, September 2021)

In this second example, the identity of the person with intellectual disability is redefined as someone who can even “teach” someone else without a disability. This is something unexpected in the field and is central to many of the theoretical developments for people with intellectual disabilities, especially the theories of Normalization and Social Role Valorization (SRV) [50]. SRV is based on this very possibility of developing social roles that are considered valuable and that are not generally associated with people with those disabilities [51,52].

Therefore, if we view QR merely as external memories, we cannot address the potential it has. What we see from this example is that the technology does not fail, even as an external memory, because it is useful, but its potential is limited if we think only from a deficit to be covered. When it is widely used in different significant spaces for the users, it becomes something to use beyond the walls of the home and allows new relationships with users to emerge. Here the person becomes autonomous, and this autonomy starts from their ability to connect, which in turn redefines what it means to be a person with intellectual disability.

In order to understand this change regarding the use of the very same technology, we turn to Michel Callon’s work on agency in the field of economic markets [32]. Callon starts from the concept of “agencement”—which has been translated to English as “assemblage”—to situate the problem of agency: who acts? 

For this author, it is always a network of socio-technical elements that are articulated, but this action can occur in different forms depending on the ways in which these elements are distributed, related, or assembled. Thus, in his development of the concept of “individual distributed agency”, Callon situates agency as the result of a concrete assemblage that can take two different forms according to two different logics, both present in the field of disability: on the one hand, a form of agency that he defines as “enabling”, which is opposed to the second, the “prosthetic”:

*“Prosthesis and habilitation are two symmetrical approaches. Both aim to compose an individual agent: the former by acting primarily on the person, the latter by striving to transform the environment. If we agree to use the proposed terminology, we can say that both compose individual agencies, but according to radically different models. Habilitation shapes an interactive agency and at the same time endows the individual with the capacity to define projects and realize them. By contrast, the addition of prostheses extends the individual to enable her to conform to common norms. The aim is to grant the individual an individual agency, but one that is disciplined. Habilitation constantly allows for the appearance of new possibilities, whereas the prosthesis limits the possible fields of action”*.

([32], p. 45)

Thus, the prosthetic mode of agency is based on the association that allows action through extensions of the body of the person with disabilities, which “equip” him or her. Hence their capabilities are restored, and the detected “disability” is compensated. That is what we can observe in the first moment, in the case of Marta and Claudia, where they do not “learn” how the dishwasher works as quickly as expected and therefore the service uses QR codes to replace their “memory”. QR codes are an extension of their body, making up for what is considered to be their deficit. This is a way of providing agency that is closely linked to the individualizing models that consider technology as an element that should replace certain shortcomings. That is, it is a prosthetic technology that allows users to be, once again, functional through a series of restorative elements, which equip and enable their autonomy [28,53].

On the other hand, in the second moment, this same QR is put in a different scenario. We see how in the case of Francisco or Elena, technology is no longer an element that seeks to individualize subjects in their actions and detach them from their dependence on the support staff. Rather, technology connects the person with intellectual disabilities with the world in a different way. The QR code, which had been viewed as a useful element inside the apartment, becomes relevant outside and opens up the possibilities for Francisco or Elena to act in other scenarios.

This is what Callon [32] defines as an enabling mode of engagement, which, unlike prosthetics, does not start from an assumed deficit or lack in the user, but takes the environment as its starting point. Here, technology acts on the outside of the disabled person and its function is not to discipline them, as we saw in the case of prosthesis, but to allow interaction and encounters, and to foster initiative in a person with a disability. 

We see how the irruption of QR post-lockdown has led to the emergence of new roles and experiences for people with learning disabilities, such as becoming technology experts in their family’s eyes or giving them the freedom to frequent bars or cafes. Here, technology opens up to a certain relationality that allows the person with intellectual disabilities an alternative way to be autonomous.

## 5. Final Remarks

This paper has shown how technologies and their implementation respond to different ways of thinking autonomy, a matter that does not depend on the quality of the material—in this case its capacity to serve as a prosthetic or to habilitate—but on the way the user relates to it [36]. That is, the same technology can serve one way or another depending on its placement in the network.

From here, and on the basis of Callons’ proposal, we have been able to see how thinking about the distribution of agency from a prosthetic perspective reveals certain limitations on the provision of care and autonomy to people with intellectual disabilities.

Prosthetic technology is coherent with the idea of autonomy being a certain capacity to make decisions, as argued from physical disability activism. As we can see in the words of Romañach, one of the promoters of the Independent Living Movement in Spain, technology should be seen as mere tools that “serve the disabled individual as their eyes, their ears or their hands”, because:

*“A person with tetraplegia, as is the case of the author of this text, may not be autonomous in performing many tasks, but is nevertheless fully capable of making decisions. The mix-up of these two concepts has led to the institutionalization of people with limited physical autonomy but full moral autonomy, who have thus been deprived of their ability to lead a life on equal terms with others, for which they are fully competent”*.

([54], p. 49)

Technology, as well as any assistance, should be nothing more than a technical substitute so that the person him/herself is able to implement their own choices. From that point of view, to be “In the hands of technology” can never be an option. Independent living activists do not accept in any way being left in the hands of others—the state, informal caregivers, or invasive technologies, among others—and therefore engage, as shown in the above quote, with a sort of “with the hands of technology”.

But this individualistic construction of what independent living implies limits the use of technologies in the field. From the parameters of the first example with Marta and Claudia, technology is only successful when it works as a simple intermediary to indicate a certain course of action and execute a certain predefined script [49]. This fails, as we have seen, because users are recalcitrant [55] in the face of a technology that limits their scope for action. Technology in this prosthetic form upholds a previous decision about what is expected to be done, and hence the service ends up failing in its attempt to use technology to replace a certain function in the network [56].

At this point, what the new take on the QR code after lockdown shows us is particularly interesting. Here technology does not encompasses pre-established courses of action, but rather it works as part of a set of precarious associations in its particular field, being viewed not so much as attending to the impairment but from the distribution of agencies, or, in words of Michel Callon [32], from the habilitation that it may allow. 

This opens up the possibility to discuss the very concept of autonomy, because the success of the QR code is not so much based on viewing autonomy as a mere individual ability to decide, but on recognizing the heterogeneous network of elements and its capacity to engage and relate to that network [24]. From here, it is no longer a technology that replaces a deficit, but a technology that opens up indeterminate possibilities that are not decided a priori. This precarious position means compromises can be found between caring and leaving room for autonomy [45], allowing the subject to be autonomous as long as he or she can relate meaningfully [57] to the world.

This is to think autonomy in a different way to the one that modernity has imposed on the political individual [58], which is the basis of prosthetic approaches to technological innovation. Here agency starts from precarious and changing associations; the possibility of thinking, acting, or deciding emerges from this entanglement of elements, human and non-human, connected in a specific moment [31]. This socio-technical assemblage needs to be constantly arranged in order to sustain autonomy [59].

Therefore, an enabling approach to technology opens the door not to thinking “in the hands” or even “with the hands” of technology, but rather to somehow position these futures “within a sociotechnical assemblage”. This means shifting away from this modern way of viewing technologies as mere tools, and instead focusing on the interrelations, on the assemblage and adjustments that allow autonomy to emerge, especially in fields where decision-making is disputed in the light of modern ways of imagining what a full citizen is.

In practice, thinking from “within a sociotechnical assemblage” can open new ways of subverting the segregation and precariousness to which people with intellectual disability are relegated [60,61], because they enable interactions and encounters in unexpected ways and places. It opens up new courses of action and enables spaces and possibilities for a collective for whom participation and public visibility are two key sociopolitical elements [50]. 

Furthermore, the work presented herein leads us to rethink the very idea of intellectual disability, whereby it is no longer a question of looking at individual attributes, but of paying attention to the links that make entanglements possible, as well as their effects on the configuration of the person with intellectual disability. Technology allows us to think about the very notion of disability, about the limits that this notion presents, and about ways of subverting these limits, which is a particularly relevant line of work with regard to care and autonomy in fields that share the same suspicion about the inability to make decisions—such as mental health or dementia. This is a cornerstone on which to pursue the use of technologies in more inclusive and relevant ways in the day-to-day lives of these people.

## Data Availability

The data presented in this study are available on request from the corresponding author due to privacy and ethical restrictions.
